# Supplementary material for: S-conLSH: alignment-free gapped mapping of noisy long reads
Source: BMC Bioinformatics. 2021 Feb 11;22:64. doi: 10.1186/s12859-020-03918-3 (PMC7879691; doi:10.1186/s12859-020-03918-3)
Supplement: Supplementary file 1 — Additional file 1. Supplementary material which contains additional results and instruction manual of the software proposed in the main article. [file 12859_2020_3918_MOESM1_ESM.pdf]

S-conLSH: Alignment-free gapped mapping of noisy long reads

Angana Chakraborty, Burkhard Morgenstern and Sanghamitra Bandyopadhyay

Supplementary Material

Contents

A Supplementary Note 1: Performance evaluation of S-conLSH in multi-threaded system 3

B Supplementary Note 2: Performance variation of S-conLSH with different values of L in multi-threaded system 7

C Supplementary Note 3: Difference between traditional spaced-word based matching algorithm and S-conLSH 8

D Instruction Manual 12

A Supplementary Note 1: Performance evaluation of S-conLSH in multi-threaded system

This section studies the behaviour of S-conLSH in a multi-threaded system. We have used a total of 146932 noisy long reads simulated from hg38 human genome using PBSIM [1], as described in the Results section of the main article, using the command "`pbsim --data-type CLR --depth 1 --length-min 1 --length-max 200000 --seed 0 --sample-fastq real.fastq hg38.fa`". The error profile has been sampled from three real human PacBio RS II P5/C3 reads listed below, concatenated as `real.fastq`.

- `m130929_024849_42213_c100518541*_s1_p0.1.subreads.fastq`
- `m130929_024849_42213_c100518541*_s1_p0.2.subreads.fastq`
- `m130929_024849_42213_c100518541*_s1_p0.3.subreads.fastq`

Table: S-1

Human simulated dataset	#Reads	#Threads	Mapper	Indexing Time (Sec)	Mapping Time (Sec)	Peak Memory Footprint (GB)
Chr#1	32290	1	Minimap2	**10**	61	1.6
			lordFAST	192	206	**1.2**
			S-conLSH	51	**38**	3.3
		4	Minimap2	**10**	25	1.8
			lordFAST	191	62	**1.2**
			S-conLSH	15	**10**	3.3
		8	Minimap2	**10**	21	1.9
			lordFAST	192	41	**1.2**
			S-conLSH	**10**	**9**	3.3

The tables [S-1]-[S-5] exhibit the performance of S-conLSH along with two other recently developed aligners Minimap2 [2] and lordFAST [3]. All methods have been executed in the same settings as mentioned in the Table 1 of the main article except running them in multiple threads. The experiment has been conducted on 5 different chromosomes of the human simulated dataset. The results obtained by single-threaded execution are also listed in the tables for the

sake of comparison. MUMmer4 [4] has been excluded from the present study due to its slow speed. The low sensitivity of Minimap [5], as observed in the Table 3 of the main article, prohibits itself from further experimentation in this section.

Table: S-2

Human simulated dataset	#Reads	#Threads	Mapper	Indexing Time (Sec)	Mapping Time (Sec)	Peak Memory Footprint (GB)
Chr#2	34309	1	Minimap2	**10**	64	1.6
			lordFAST	170	216	**1.2**
			S-conLSH	54	**44**	3.4
		4	Minimap2	**10**	47	1.8
			lordFAST	170	67	**1.2**
			S-conLSH	16	**12**	3.4
		8	Minimap2	**10**	22	1.9
			lordFAST	170	46	**1.2**
			S-conLSH	**10**	**10**	3.4

Table: S-3

Human simulated dataset	#Reads	#Threads	Mapper	Indexing Time (Sec)	Mapping Time (Sec)	Peak Memory Footprint (GB)
Chr#3	28109	1	Minimap2	**8**	52	1.4
			lordFAST	135	167	**1.1**
			S-conLSH	45	**30**	3.1
		4	Minimap2	**8**	21	1.5
			lordFAST	135	44	**1.1**
			S-conLSH	13	**9**	3.1
		8	Minimap2	**8**	17	1.7
			lordFAST	135	28	**1.1**
			S-conLSH	9	**8**	3.1

The table S-1 describes the indexing time, mapping time and the peak memory footprint of the three different methods on 32290 different reads simulated from the chromosome-1 of the human genome. The best results in each category have been marked as bold. Please note that the sensitivity and precision

values of alignment quality have not been reported in the present study as they remain unchanged with the degree of parallelism. Please refer to the Table 3 of the main article for detailed comparison of sensitivity and precision of the alignments produced by different methods.

Table: S-4

Human simulated dataset	#Reads	#Threads	Mapper	Indexing Time (Sec)	Mapping Time (Sec)	Peak Memory Footprint (GB)
Chr#4	26871	1	Minimap2	**8**	51	1.4
			lordFAST	129	158	**1.0**
			S-conLSH	41	**29**	3.0
		4	Minimap2	**8**	20	1.5
			lordFAST	129	42	**1.0**
			S-conLSH	12	**9**	3.0
		8	Minimap2	**8**	18	1.7
			lordFAST	129	27	**1.0**
			S-conLSH	**8**	**8**	3.0

It can be observed from table S-1 that S-conLSH is always the fastest in terms of mapping time when experimented with different number of threads. The S-conLSH indexer, on the other hand, is getting faster with the increase of parallelism and matches up with the speed of Minimap2 when run in 8 parallel threads.

Table: S-5

Human simulated dataset	#Reads	#Threads	Mapper	Indexing Time (Sec)	Mapping Time (Sec)	Peak Memory Footprint (GB)
Chr#5	25353	1	Minimap2	**7**	48	1.4
			lordFAST	123	149	**0.9**
			S-conLSH	39	**23**	2.9
		4	Minimap2	**7**	26	1.4
			lordFAST	123	40	**0.9**
			S-conLSH	12	**8**	2.9
		8	Minimap2	**7**	16	1.6
			lordFAST	123	25	**0.9**
			S-conLSH	**7**	**6**	2.9

Note that, Minimap2 and lordFAST use multiple threads only at the time of read-alignment. The indexing time of these two methods, therefore, does not change with the number of threads. A similar scenario is visible in the results of 4 other chromosomes of the human simulated dataset (refer tables [S-2] to [S-5]).
Although slower in terms of mapping and indexing time, lordFAST has the smallest memory footprint among the three. The memory load of S-conLSH is higher than the other two methods. This is because S-conLSH index stores more information to ensure the highest level of sensitivity of the read-mapping without performing an actual base-by-base alignment. Hence, it can be concluded that S-conLSH improves its rum-time performance by taking advantage of the parallelism while keeping the quality of mapping at its best.
It is interesting to observe that the memory requirement of Minimap2-aligner (as Minimap2-indexer does not work in multiple threads) increases with the increase of thread count. The same of the other two methods, S-conLSH and lordFAST, does not change significantly with higher degree of threading.

B Supplementary Note 2: Performance variation of S-conLSH with different values of L in multi-threaded system

To study the performance variation of S-conLSH with different values of L in a multi-threaded system, we have conducted an experiment on 32290 reads of chromosome1 of human simulated dataset. S-conLSH has been executed in 4 concurrent threads for 5 different values of L. The results are summarised in Table S-6. The results obtained for the default value, i.e., $L = 2$ of S-conLSH have been marked as bold. Note that the other parameter values are fixed at the default ($K = 2$, context size $(2\lambda + 1) = 7$, and $z = 5$) settings.

Table S-6

Human simulated dataset	#Reads	#Threads	Mapper	L	Indexing Time (Sec)	Mapping Time (Sec)	Peak Memory Footprint (GB)
Chr#1	32290	4	S-conLSH	1	15	8	3.31
				**2**	**15**	**10**	**3.31**
				5	16	52	3.316
				7	15	91	3.319
				10	15	143	3.32

It can be observed from Table S-6 that the mapping time increases with L. This is because, L indicates the number of different hash tables (one for each of the L different spaced-seeds) used to compute the mapping of noisy long reads. Therefore the search becomes more rigorous, with the increased values of L, having considered all spaced-contexts obtained from L different patterns. This is, however, useful for highly sensitive applications at the expense of a few more seconds of mapping time. The indexing time, on the other hand, does not vary with the change in L. A similar conclusion has been made from the Table 6 of the main article. In spite of the fact that S-conLSH is executed in 4 different threads, the increase in L does not add up significantly to the memory footprint. Hence, it can be concluded that S-conLSH may be used with higher values of L, being distributed over multiple concurrent threads, without requiring much memory space.

C Supplementary Note 3: Difference between traditional spaced-word based matching algorithm and S-conLSH

This section elaborates the difference of S-conLSH over the standard spaced-word based algorithms[6, 7, 8, 9]. The spaced-seeds or patterns used in this illustration can be found at Fig.S.1(a). The Fig.S.2 describes the matching using traditional spaced-word based algorithms with the same strings and patterns used in Fig.S.3 for S-conLSH. Note that Fig.S.3 is exactly the same as Figure 2 of the main article which is repeated here for better understanding.

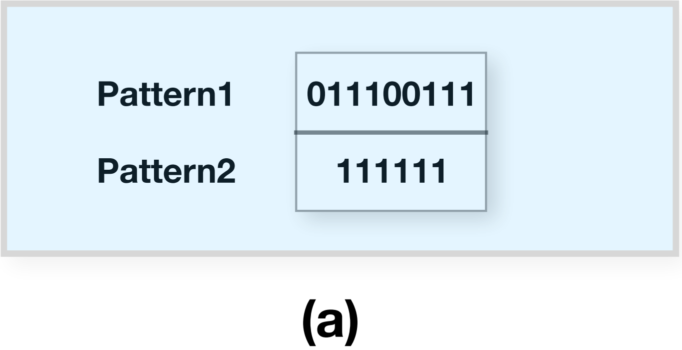

Figure S.1: The set of spaced-seeds used for illustration.

In the standard spaced word based methods one or multiple spaced-seeds or patterns are used to find matching between the sequences. A base-by-base matching is required in the sequences corresponding to the positions of '1's in the pattern. The positions of '0's are considered as don't care locations. If two sequences have the same bases in the positions of '1's in the pattern, then it is a hit. The matches or mismatches whatever there are in the position of '0's are simply ignored. Fig.S.2 shows an example of this using two strings "ATTCGGTAA" and "TTCTAAGTA" (same strings which have been used in Figure 2 in the main article).

It can be seen from the Fig.S.2(b) that strings "ATTCGGTAA" and "TTCTAAGTA" do not make a hit as they have different bases (marked as red in the figure) at the positions of '1's in the pattern "01100111". It is similar for the second pattern "111111" as well (see Fig.S.2(c)). Therefore, both the patterns identify these two

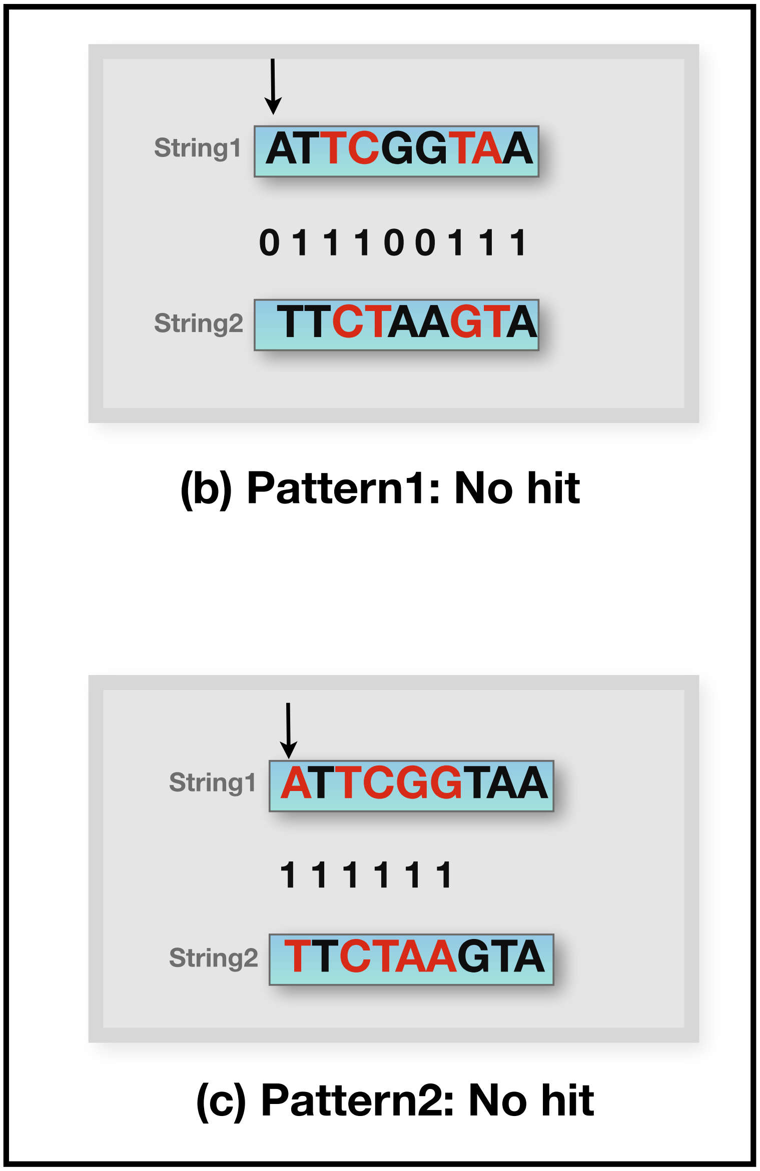

Figure S.2: Standard spaced-word based matching using the patterns described in Fig. S.1(a).

strings as dissimilar according to the standard spaced-word based methods.

However, a different scenario has been experienced when we used the same

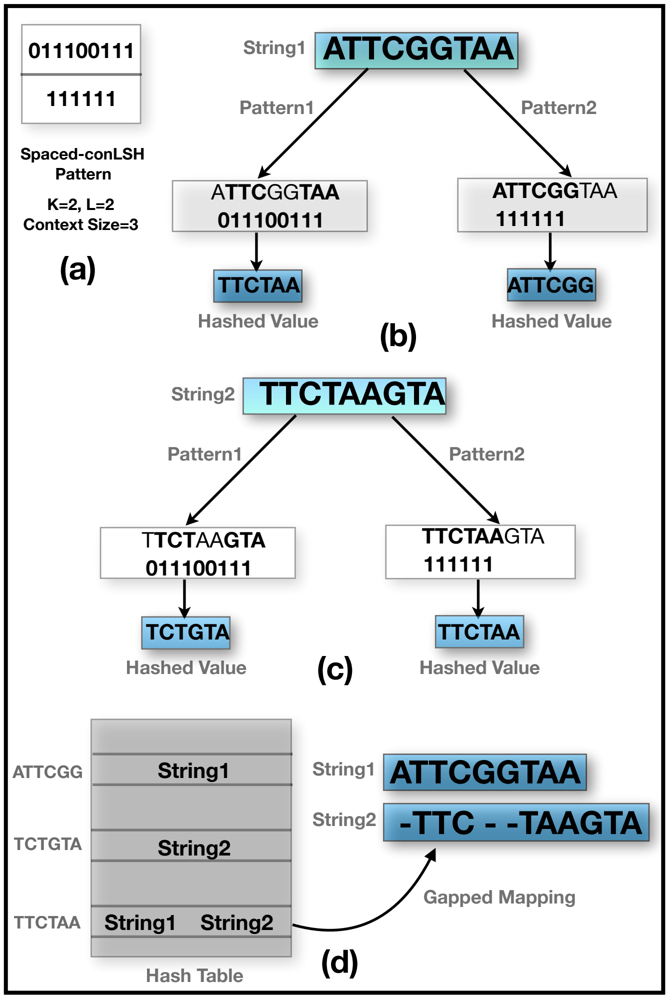

Figure S.3: A schematic illustration of gapped-mapping using S-conLSH on "ATTCGGTAA" and "TTCTAAGTA" respectively using the patterns of Fig.S.1(a).

patterns on the strings "ATTCGGTAA" and "TTCTAAGTA" in S-conLSH (please see Fig.S.3). S-conLSH computes the spaced-contexts (see Definition 4 in the main article) or hashed values from the strings "ATTCGGTAA" and "TTCTAAGTA" using both the patterns "01100111" and "111111" (depicted in Fig.S.3(b) and (c)). Then, the strings are inserted in a hash table corresponding to their hashed values (Fig.S.3(d)). It can be seen that String1 and String2 are inserted together in hash index "TTCTAA". This is because they have produced the same hash value, "TTCTAA", for pattern1 and Pattern2 respectively. As they are hashed

together in the same slot of the hash table, S-conLSH identifies them as similar. This results in a mapping between String1 and String2 introducing gaps in the positions of '0's in the corresponding pattern. The gapped mapping is obtained only because S-conLSH hashes the spaced-contexts obtained from different patterns together where each pattern has different distribution of '0's and '1's, generated using Algorithm 1 described in the main article.

Please note that in the figures, the patterns have been shown to be placed only at the first positions of the strings for the sake of simplicity. In the actual computation of spaced-context values, each pattern slides over the strings starting from the first position (as shown in the Fig.S.4) and the hashed values from all the positions are considered.

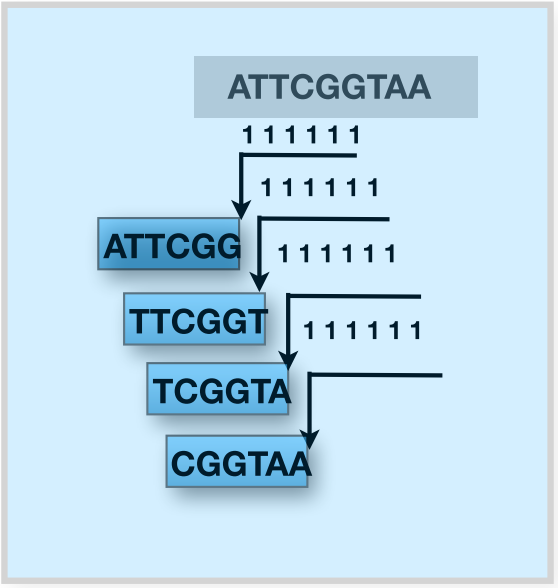

Figure S.4: A schematic illustration of sliding patterns over the length of the string to compute the hashed values.

D Instruction Manual

Spaced context based Locality Sensitive Hashing, S-conLSH-2.0, is a new mapper (`https://github.com/anganachakraborty/S-conLSH-2.0.git`) that facilitates gapped mapping of noisy long reads to the corresponding target locations of a reference genome, with multiple spaced patterns. It can handle multiple simultaneous threads to achieve better run-time performance. We have examined the performance of the proposed method on 5 different real and simulated datasets. S-conLSH is at least 2 times faster than the recently developed state-of-the-art aligner lordFAST [3]. It achieves a sensitivity of 99%, without using any traditional base-to-base alignment, on human simulated sequence dataset. By default, S-conLSH provides an alignment-free mapping in PAF format. If a base level alignment is required, S-conLSH provides an option (--align 1) to generate alignment in SAM format using ksw library (`https://github.com/attractivechaos/klib`).

Installation

Current version of S-conLSH needs to be run on the Linux operating system. The source code is written in C++. The makefile is attached. Use make command to generate the executables. The binary 'S-conLSH' performs indexing of the reference genome and then aligns the long and noisy PacBio reads to it.

OpenMP support is required to execute S-conLSH in multiple threads. Run "sudo apt-get install libomp-dev" in Linux terminal to install it. The default settings of the method work in a single-threaded version. However, it can be executed in multiple threads using the option "--thread". Multi-threaded execution incurs higher CPU and memory load. A good choice of selecting the number of threads would be equal to the number of CPU-cores available.

Synopsis

S-conLSH <PathOfSourceFiles> <ReferenceGenome> <ReadFile> [-K concatenationFactor] [-L NumberOfHashTables] [--lambda contextFactor] [--zero spacesInPatterns] [-w windowsHits] [-m candidates] [-x match] [-y mismatch] [-q gapOpen] [-r gapExtension] [-a alignInSAM] [--thread numberOfThreads] > <OutputFile>

Quick start

The package includes sample reference genome and SMRT reads to demonstrate a quick start guide.

For alignment free mapping in PAF format
./S-conLSH ../src/ ../sample_data/ecoli_AE005174v2.fas ../sample_data/SRR801638.fasta > sample.paf

For SMRT alignment in SAM format
./S-conLSH ../src/ ../sample_data/ecoli_AE005174v2.fas ../sample_data/SRR801638.fasta –align 1 > sample.sam

Parameters

-K, --K	<int>	Concatenation factor of locality sensitive hashing [Default=2]
-L, --L	<int>	Number of hash tables in conLSH framework [Default=2]
--lambda	<int>	The context factor [Default=3]
--zero	<int>	The number of don't cares or zeros in the S-conLSH pattern [Default=5]
-w, --window-hits	<int>	The max allowed number of windows hitting by a k-mer [Default=1000]
-m, --candidates	<int>	The number of candidates for extension [Default=400]
-x, --match	<int>	Score of match for the alignments in extension phase [Default=2]
-y, --mismatch	<int>	Mismatch penalty for the alignments in extension phase [Default=5]
-q, --gap-open	<int>	Gap open penalty for the alignments in extension phase [Default=2]
-r, --gap-extension	<int>	Gap extension penalty for the alignments in extension phase [Default=1]
-a, --align	<int>	Value=1, outputs alignment in SAM format [Default=0, Alignment-free PAF format output]
--thread	<int>	Number of threads to be forked in multi-threaded system [Default=1]
-h, --help		Help

References

[1] Ono, Y., Asai, K., Hamada, M.: Pbsim: Pacbio reads simulator–toward accurate genome assembly. Bioinformatics **29**(1), 119–121 (2012)

[2] Li, H.: Minimap2: pairwise alignment for nucleotide sequences. Bioinformatics **34**(18), 3094–3100 (2018)

[3] Haghshenas, E., Sahinalp, S.C., Hach, F.: lordfast: sensitive and fast alignment search tool for long noisy read sequencing data. Bioinformatics **35**(1), 20–27 (2018)

[4] Marçais, G., Delcher, A.L., Phillippy, A.M., Coston, R., Salzberg, S.L., Zimin, A.: Mummer4: A fast and versatile genome alignment system. PLoS Computational Biology **14**(1), 1005944 (2018)

[5] Li, H.: Minimap and miniasm: fast mapping and de novo assembly for noisy long sequences. Bioinformatics **32**(14), 2103–2110 (2016)

[6] Ma, B., Tromp, J., Li, M.: Patternhunter: faster and more sensitive homology search. Bioinformatics **18**(3), 440–445 (2002)

[7] Li, M., Ma, B., Kisman, D., Tromp, J.: PatternHunter II: Highly sensitive and fast homology search. Genome Informatics **14**, 164–175 (2003)

[8] Hahn, L., Leimeister, C.-A., Ounit, R., Lonardi, S., Morgenstern, B.: *rasbhari*: optimizing spaced seeds for database searching, read mapping and alignment-free sequence comparison. PLOS Computational Biology **12**, 1005107 (2016)

[9] Leimeister, C.-A., Boden, M., Horwege, S., Lindner, S., Morgenstern, B.: Fast alignment-free sequence comparison using spaced-word frequencies. Bioinformatics **30**(14), 1991–1999 (2014)
